# Tendinopathy alters the time course of free achilles tendon morphological recovery following eccentric exercise

**DOI:** 10.1038/s41598-026-55189-2

**Published:** 2026-06-10

**Authors:** Leila Nuri, Steven J. Obst, Rod S. Barrett

**Affiliations:** 1 Center for Graduate Studies, Department of Physical Therapy, West Coast University, Los Angeles, CA USA; 2 School of Health, Medical and Applied Sciences, College of Health Sciences, Central Queensland University, Bundaberg, QLD Australia; 3 School of Allied Health, Sport and Social Work, Griffith University, Gold Coast, QLD Australia; 4 Australian Centre for Precision Health and Technology (PRECISE), Griffith University, QLD Gold Coast, Australia

**Keywords:** Anatomy, Medical research

## Abstract

The purpose of this study was to investigate the immediate and short-term (0–3 h) effects of a single bout of eccentric plantarflexion exercise on three-dimensional (3D) Achilles tendon (AT) morphology in healthy and tendinopathic tendons. The eccentric exercise protocol consisted of six sets of 15 unilateral heel drops performed on a step. Freehand 3D ultrasound scans of the AT were acquired immediately before exercise and at 30-minute intervals over a 3-hour post-exercise recovery period to quantify tendon volume, length, and cross-sectional area (CSA). An exponential function characterized the recovery time course and calculated recovery time constants. Following eccentric exercise, both tendinopathic and healthy tendons exhibited immediate reductions in volume and CSA and increased tendon length. Tendinopathic tendons demonstrated a faster initial recovery of morphological properties, as indicated by shorter recovery time constants, compared with healthy tendons. However, this early recovery plateaued after one hour, and morphological recovery remained significantly lower than that of healthy tendons during the 2–3 h post-exercise period. These findings suggest that tendinopathic tendons exhibit an early but incomplete morphological recovery characterized by faster initial fluid reabsorption followed by a reduced capacity for sustained recovery, likely reflecting degeneration-related alterations in tendon structure and poroelastic behavior.

**Supplementary Information:**

The online version contains supplementary material available at 10.1038/s41598-026-55189-2.

## Introduction

The Achilles tendon (AT) transmits force generated by the gastrocnemius and soleus muscles to the calcaneus^1^ and exhibits both elastic (solid-like) and viscous (fluid-like) behavior^2^. The mechanical function of the AT is modulated by the interplay between collagen fiber deformation and fluid movement within the tendon^3^, and allows the AT to withstand substantial tensile forces and associated longitudinal strains during dynamic activities such as walking, running, and jumping^4,5^. During mechanical loading, increased intratendinous hydrostatic pressure^6^ can lead to time-dependent fluid flow, resulting in the redistribution of fluid within the tendon core and/or exudation toward the surrounding sheath or peri-tendinous space^7,8^. Such physiological responses to loading are reflected in alterations to the AT macromorphology^9,10^. For instance, fluid exudation can reduce tendon volume and cross-sectional area (CSA), whereas internal fluid redistribution alters regional fluid content without changing overall volume^10^. Changes in AT volume, CSA, and length in response to mechanical loading in healthy tendons are reported to return to baseline levels following a period of unloading^11,12^. In contrast to healthy tendons, prior experimental and modeling studies indicate that degeneration-related changes in tendon structure (e.g., collagen disorganization, matrix degradation) are associated with increased fluid mobility and altered intratendinous pressure responses under load^6,13^. Further, in vivo studies report that tendinopathic ATs experience greater load-induced reductions in tendon CSA, volume, and thickness compared to healthy ATs^14^. However, compared with healthy ATs, relatively little is known about the time course of morphological recovery in tendinopathic ATs following mechanical loading. Investigating the immediate morphological responses of the AT and its recovery following an acute loading bout in both healthy and tendinopathic tendons may improve understanding of pathological tendon mechanical behavior and its underlying mechanisms. The early recovery phase following loading is associated with rapid (exponential) fluid redistribution and visco-poroelastic alterations within the tendon matrix^7,11^, processes that may be altered in tendinopathic tissue. Therefore, examining this early post-loading period (minutes to hours) may provide insight into pathological tendon mechanics and help inform load management during rehabilitation and daily activities in the immediate recovery phase.

Eccentric exercise is widely recognized as an effective conservative intervention for the management of Achilles tendinopathy^15,16^. Although heavy slow resistance training has also been shown to be effective in the treatment of Achilles tendinopathy^17^, eccentric loading remains one of the most widely studied and commonly implemented rehabilitation approaches for Achilles tendinopathy and has frequently been used in experimental studies investigating tendon responses to mechanical loading^18–20,24^. In vivo studies examining the acute effects of eccentric exercise on healthy tendons have reported immediate post-exercise reductions in tendon CSA and thickness at rest^18,19^ and during contraction^20^. The magnitude of these changes is influenced by loading speed and intensity^18,21^. Complementary ex vivo investigations using cyclic loading paradigms designed to replicate eccentric strain profiles during exercise demonstrate rapid tendon deformation, fluid exudation, and collagen fiber reorganization, with the magnitude, frequency, and duration of strain modulating these responses^8,22,23^. Despite the clinical efficacy of eccentric exercise, only one in vivo study has directly examined the immediate morphological response of tendinopathic ATs to an acute bout of eccentric exercise, reporting an immediate post-exercise reduction in tendon thickness^24^. Comparative investigations of the acute morphological response between tendinopathic and healthy tendons post–eccentric exercise are also limited. Ho and Kulig^25^ observed similar reductions in water content in both tendinopathic and healthy patellar tendons following eccentric exercise, whereas Grigg et al.^24^ reported a smaller reduction in thickness strain in tendinopathic ATs compared with healthy controls, suggesting altered fluid dynamics in pathological tissue. To date, no study has compared the immediate three-dimensional (3D) morphological changes (i.e., volume, length, and CSA) of tendinopathic and healthy ATs following an acute bout of eccentric exercise.

Evidence suggests that the time course of AT recovery is strongly influenced by loading magnitude, exercise type, and the specific morphological or mechanical parameter assessed. Prolonged or high-demand activities, such as cross-country running or elite Australian Football League competition, have been associated with delayed recovery of AT volume and stiffness, requiring up to 72–96 h in healthy individuals^12,26^. In contrast, lower-intensity or controlled loading protocols allow more rapid recovery, with AT thickness returning within 24 h after low-intensity plantar-flexion exercise without blood-flow restriction^27^, and AT length and CSA recovering within two hours following a submaximal conditioning protocol^11^. Grigg et al.^19^ and Wearing et al.^28^ investigated recovery of healthy AT thickness following eccentric loading and reported full restoration of tendon thickness by 24 h. In both studies, recovery followed an exponential pattern, with recovery time constants of approximately 2.5 h and 6.5 h, respectively. Full recovery in AT thickness in tendinopathic ATs following eccentric exercise was also reported at 24 h^24^. However, no study to date has directly compared the 3D morphological recovery of healthy and tendinopathic tendons following an acute bout of eccentric exercise. Conventional two-dimensional (2D) ultrasound techniques typically quantify tendon thickness or CSA at a single anatomical location and therefore cannot capture 3D changes in tendon structure. In contrast, freehand 3D ultrasound enables reconstruction of the entire free tendon, allowing simultaneous assessment of tendon volume, length, and CSA along its length. Quantification of tendon volume allows differentiation between tendon fluid exudation (fluid leaving the tendon) and fluid redistribution (fluid movement within the tendon) during loading and recovery^14,20^.

The purpose of this study was to investigate the immediate and short-term (0–3 h) effects of a single bout of eccentric heel-drop exercise on the 3D morphology (i.e., volume, length, and CSA) of healthy and tendinopathic ATs. Based on previous evidence suggesting greater fluid mobility and altered intratendinous pressure responses in pathological tendon, we hypothesized that tendinopathic tendons would exhibit reduced restoration of tendon morphology during the recovery period following eccentric exercise compared with healthy tendons.

## Materials and methods

### Participants

Ten male patients with unilateral mid-portion Achilles tendinopathy were included in this study (age: 41.2 ± 10.3 years; physical activity: 5457 ± 2027 MET-min.week^− 1^). Eligibility for inclusion was evaluated by an experienced musculoskeletal physical therapist. The diagnosis was based on a combination of clinical history (activity-related Achilles pain and morning stiffness), physical examination (localized tendon pain and thickening), and ultrasound imaging findings, including increased tendon thickness and focal hypoechogenicity^14,29^. Inclusion criteria were male adults aged 18–60 years with unilateral symptomatic mid-portion Achilles tendinopathy, presence of symptoms for more than three months (4.2 ± 1.9 years), and a Victorian Institute of Sports Assessment-Achilles tendon (VISA-A) score (0–100) of less than 80 points^14,30^(60.2 ± 5.4). Patients presenting with insertional tendinopathy, bilateral tendinopathy, a history of AT partial tear, surgery, rupture, or calf muscle injury were excluded^14,30^. Additionally, ten control male participants from the local university community, matched to the patients with Achilles tendinopathy by gender, age (42.2 ± 9.4 years), and physical activity (5862 ± 2079 MET-min.week^− 1^), were included in the study. Ethical approval was obtained from the Griffith University Human Research Ethics Committee (protocol no. 2012/434), and the study was conducted in accordance with the Declaration of Helsinki. All participants provided written informed consent prior to participation. The sample size was comparable to previous in vivo ultrasound studies examining tendon morphological responses to mechanical loading, which typically include approximately 8–12 participants per group due to the time-intensive imaging procedures and detailed post-processing required^14,24,29,30,37^.

### Experimental protocol

Participants were instructed to lie prone on an examination plinth with their knee and hip fully extended and the ankle joint in a neutral position. The foot was securely affixed to a footplate using a custom-designed adjustable ratchet system to ensure standardized ankle positioning across participants^11,14^. During testing, participants initially performed several sub-maximal isometric plantarflexion contractions to precondition the tendon^30,31^. Resting ultrasound scans of the free AT were obtained: one immediately before the eccentric exercise protocol and the other immediately post-exercise, followed by additional scans during recovery at 30-minute intervals up to 3 h post-exercise. Participants were instructed to use the restroom before the experiment began. During the 3-hour recovery period, participants remained seated between measurements and were asked to avoid weight-bearing activities to minimize additional loading of the AT.

**Fig. 1:**
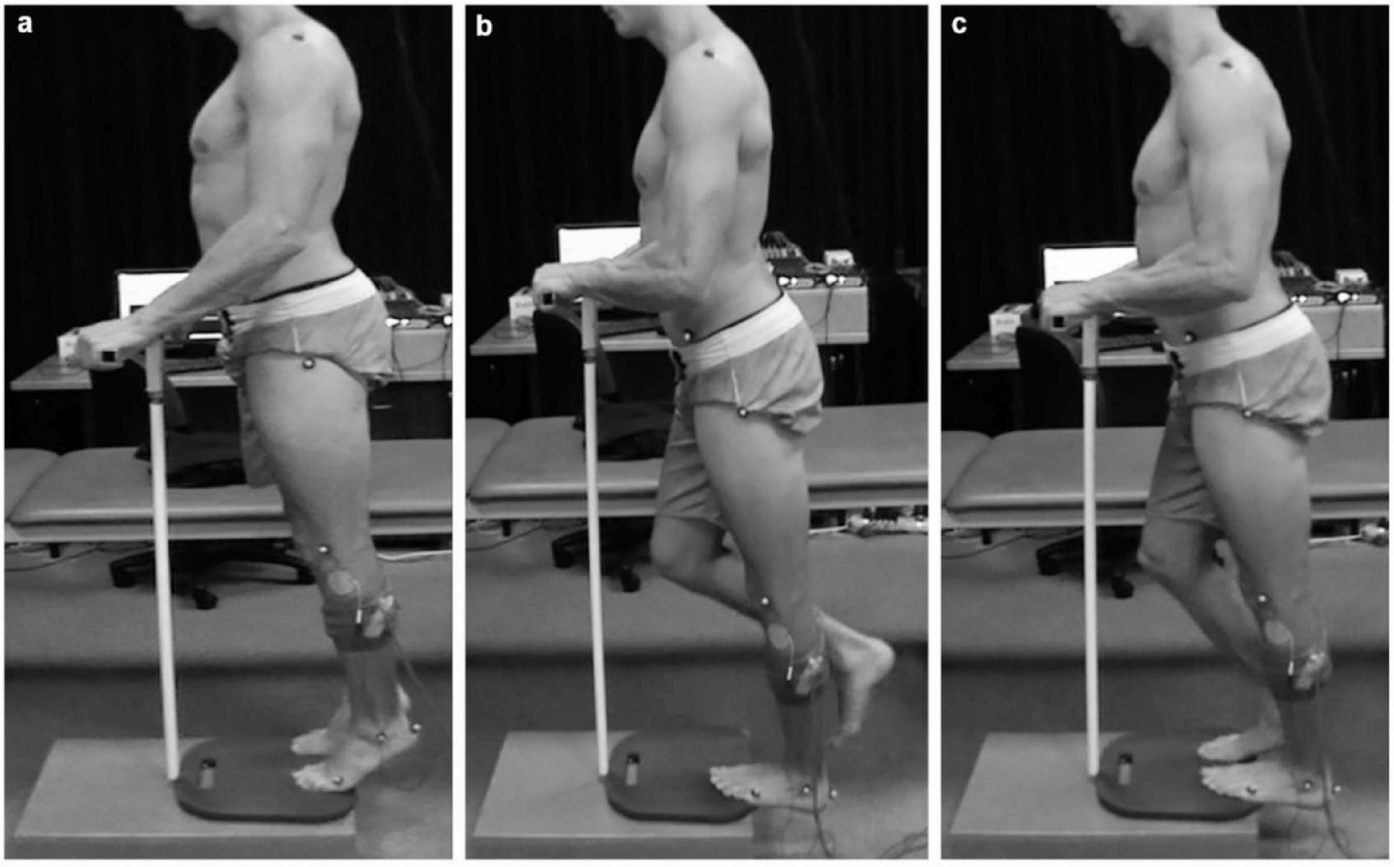
Eccentric heel-drop exercise protocol.(**a**) Starting position: the participant stands with the forefoot of the tendinopathic limb placed on the edge of a custom-made step, with the ankle in maximal plantar flexion. (**b**) Eccentric phase: the heel of the tendinopathic limb is slowly lowered below the level of the forefoot to achieve maximal dorsiflexion while maintaining a straight knee. (**c**) Return phase: the contralateral healthy limb is placed on the step and used to return the body to the initial position (concentric phase).

For the eccentric exercise protocol, participants were instructed to position the forefoot of their tendinopathic leg on the edge of a custom-made step, ensuring their ankle was in maximal plantar flexion [Figure 1A]. The exercise was performed using body weight only, with no additional external load applied. Eccentric loading was accomplished as participants lowered their heel below the forefoot level to achieve maximal dorsiflexion while maintaining a straight knee [Figure 1B]. Subsequently, the forefoot of the contralateral healthy limb was placed on the step, and all weight was shifted to this limb, which was then used to return the body to the initial position (i.e., concentric phase) [Figure 1C]^32^. This protocol was repeated for six sets, each comprising fifteen repetitions, with a one-minute rest interval between sets. The eccentric phase of the exercise was required to be completed in three seconds at a constant speed, monitored via the Pro Metronome app displayed on a computer screen for audio-visual feedback. The healthy control group performed the eccentric exercise with a randomly assigned healthy leg. Participants were instructed to perform the exercise protocol barefoot to optimize ankle range of motion. All participants received comprehensive instructions on the exercise protocol from a physical therapist and practiced under her supervision prior to testing. To verify that the eccentric loading protocol did not differ between groups, the movement was recorded and analyzed using 2D video analysis (Kinovea, version 2025.1.1, www.kinovea.org). Ankle joint range of motion (ROM) during the eccentric phase was calculated as the difference between maximal plantarflexion and maximal dorsiflexion angles. Angular velocity was estimated as the ratio of ankle angular displacement (ROM) to the duration of the eccentric phase.

### Freehand 3D-ultrasound system

The freehand 3D ultrasound system consisted of a 2D B-mode ultrasound device (SonixTouch; Ultrasonix, Richmond, BC, Canada) and a five-camera optical tracking system (OptiTrack V100:R2, Tracking Tools Version 2.5.2, NaturalPoint, Corvallis, OR, USA). This tracking system records the position and orientation of the transducer during scanning by monitoring four reflective markers rigidly attached to the transducer^33^. The obtained B-mode images were transformed into the global coordinate system using the Stradwin software package (v6.03, Mechanical Engineering, Cambridge University, Cambridge, UK; https://mi.eng.cam.ac.uk/Main/StradWin) to construct a 3D image of the free AT. The temporal and spatial calibration of the ultrasound transducer was performed in a water bath (25 °C) using a single-wall phantom calibration method, in accordance with the Stradwin software guidelines^34^. Post-calibration, the coordinates of any pixel within a 2D ultrasound image were converted into 3D space with an error margin of less than ± 0.4 mm^35^. The ultrasound scanning was conducted with a 58 mm linear transducer (L14-5 W/60 linear, Ultrasonix), utilizing the following image settings: frame rate of 60 Hz, depth of 40 mm, gain at 50%, dynamic range of 65 dB, map setting of 4, and power setting of 0. Images of the free AT were subsequently acquired using a single transverse sweeping scan by manually moving the ultrasound transducer from the distal calcaneus to the soleus muscle-tendon junction (SOL MTJ) at a steady speed of approximately 7 mm/s. The total scanning duration was approximately 10 s, resulting in stacks of 2D B-mode images comprising roughly 600 frames, with an inter-frame distance of approximately 0.1 mm. The resting ultrasound scan of free AT was performed twice at each time point, and the scan with the best image quality was selected for image analysis.

### 3D ultrasound image analysis

**Fig. 2:**
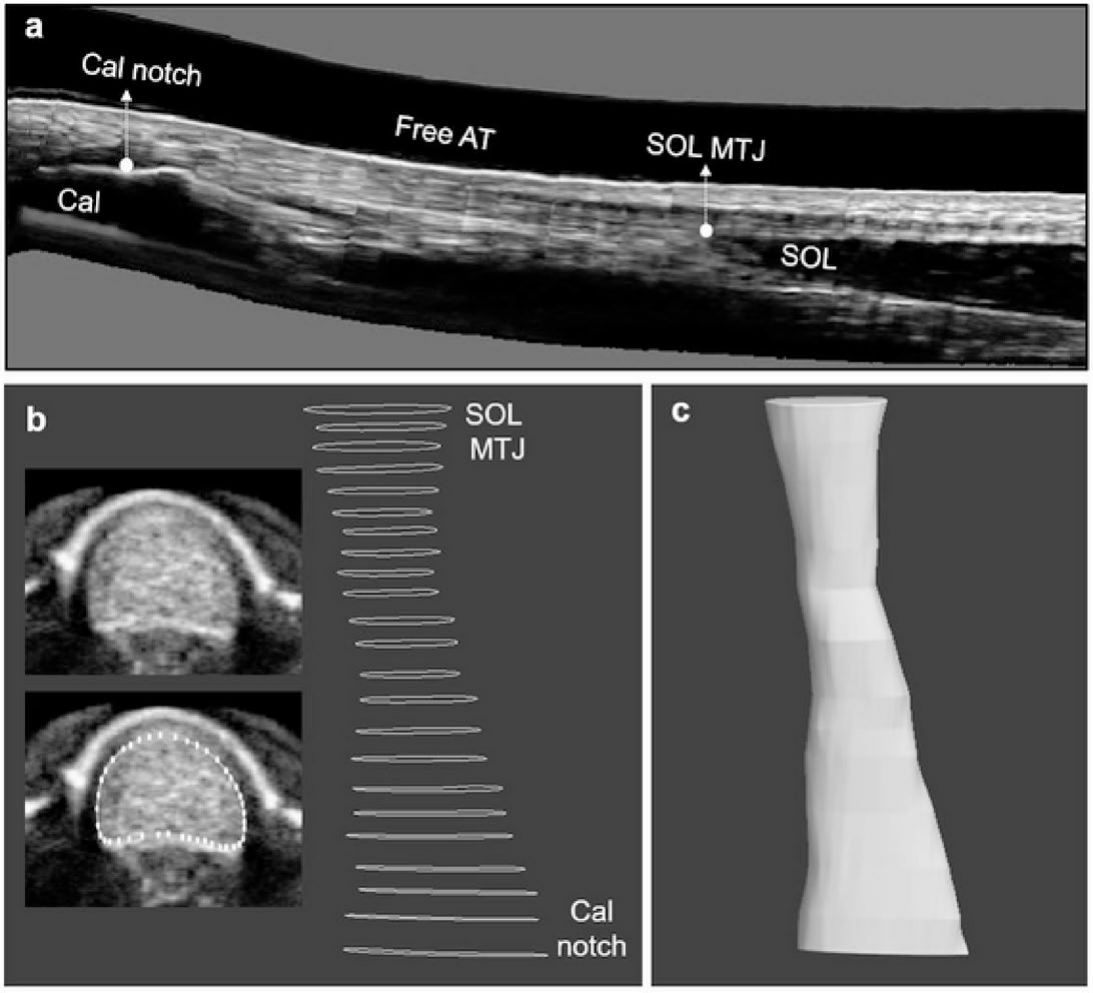
Measurement of free Achilles tendon (AT) length, cross-sectional area (CSA) segmentation, and volume reconstruction from a representative participant. (a) Sagittal plane re-slices of a reconstructed three-dimensional (3D) ultrasound image of the free AT. Two anatomical landmarks [calcaneal (Cal) notch and soleus muscle–tendon junction (SOL MTJ)] were identified and segmented on 3D AT images to determine tendon length. (**b**) Representative transverse ultrasound image of the AT showing the manual digitization of the CSA. The CSA of the free AT was manually digitized from transverse images at 2 mm intervals extending from the Cal notch to the SOL MTJ. (**c**) The 3D volume of the free AT was reconstructed from the segmented CSAs using a surface interpolation algorithm implemented in Stradwin software (v6.03, https://mi.eng.cam.ac.uk/Main/StradWin).

All 3D-US images were segmented using Stradwin software. Two anatomical landmarks (calcaneal notch and SOL MTJ) were identified on sagittal and transverse images, and subsequently marked manually using the 3D landmark tool. The Free AT length was defined as the point-to-point distance between the calcaneal notch and SOL MTJ Figure 2A. Free AT cross-sections were manually outlined from the transverse B-mode images, extending from the calcaneal notch to the SOL MTJ at 2 mm intervals Figure 2B. The reconstruction of the free AT volume was performed on the segmented cross-sections using an integrated 3D rendering algorithm within the Stradwin software Figure 2C. The mean free AT CSA (mm^2^) at each measurement time point (i.e., Pre, 0, 0.5, 1, 1.5, 2, 2.5, and 3 h) was determined by dividing free AT volume by the free AT length (×1000). Absolute changes in tendon volume, length, and CSA, were computed by subtracting the values at each time point from the corresponding pre-exercise values. Relative changes in tendon volume, length, and CSA at each time point were then calculated as the ratio of the absolute values to the corresponding pre-exercise values, expressed as a percentage.

The intra-rater reliability of tendon length and CSA measurements obtained using the same freehand 3D ultrasound protocol has previously been evaluated for the same investigator. Excellent reliability was observed, with intraclass correlation coefficients greater than 0.98 for tendon length and 0.97 for CSA measurements, indicating very high measurement reproducibility^31^.

### Statistical analysis

Data were available for *n* = 10 tendinopathic and *n* = 10 healthy tendons (total *n* = 20). Independent two-tailed t-tests were used to compare the free tendon resting dimensions (i.e., volume, length, CSA) between groups (tendinopathy and healthy). Independent t-tests were also used to compare body mass, ankle ROM, and angular velocity during the eccentric exercise between groups to confirm comparable loading conditions. Data normality was assessed using the Shapiro–Wilk test, and visual inspection of residuals confirmed approximate normal distributions for all dependent variables. Two-way full factorial repeated measures general linear models (2 × 8) were used to examine the effects of tendon type (tendinopathy and healthy) and measurement time points (pre-exercise, 0, 0.5, 1, 1.5, 2, 2.5, and 3 h) on the percentage change in free AT volume, length, and CSA separately, with measurement time point treated as the within-subject factor. Sphericity was assessed using Mauchly’s test, and Greenhouse–Geisser corrections were applied where appropriate. Planned contrasts (a priori, implemented using SPSS CONTRAST syntax) compared tendon percentage change values at each post-exercise measurement time point to both pre-exercise values and the values from the immediately preceding measurement time point for each tendon, as well as between tendinopathic and healthy tendons at each post-exercise measurement time point. Contrasts were specified a priori; therefore, no additional adjustment to α was applied. The significance level was set at α = 0.05. Effect sizes were reported as Cohen’s d for independent t-tests and partial eta squared (ηp²) for ANOVA. All analyses were performed using the Statistical Package for the Social Sciences (SPSS) version 29 (IBM Corp., Armonk, NY, USA). In the text, data are reported as means ± standard deviation (SD) and in the figures, they are presented as means ± standard error of the mean (SEM).

The recovery of free AT morphological properties over the recovery period was modeled using an exponential function based on the Kelvin-Voigt model of viscoelasticity^36^ as follows:

$$[f\left( t \right)\text{ }=\text{ }a\text{ }\times \text{ }\left( 1\text{ }-\text{ }\exp \left( -t/b \right) \right)\text{ }+\text{ }c$$

where *a*, *b*, and *c* are coefficients, *t* represents time. The function’s fit to the data was assessed using the root mean square error (RMSE) and the coefficient of determination (R²). The time constant (b) for the recovery of free tendon morphology following eccentric exercise is defined as the time required for the tendon to reach 63% of its pre-exercise value in these parameters^36^.

## Results

### Participant characteristics and exercise execution

Body mass and movement characteristics during the eccentric heel-drop exercise were comparable between groups. The mean ankle ROM during the eccentric phase was 38.55 ± 9.60° in the tendinopathy group and 36.61 ± 10.82° in the healthy group, with no significant difference between groups (t(18) = −0.42, *p* = 0.91). The mean angular velocity was 12.49 ± 3.17°/s in the tendinopathy group and 12.02 ± 3.41°/s in the healthy group, also with no significant difference (t(18) = −0.31, *p* = 0.98). The mean body mass was 81.10 ± 6.15 kg in the tendinopathy group and 79.29 ± 7.61 kg in the healthy group, with no significant difference between groups (t(18) = −0.40, *p* = 0.11), indicating that comparable mechanical loading was applied during the exercise protocol.

### Effects of tendon type and measurement time points on tendon volume, length, and CSA

**Fig. 3:**
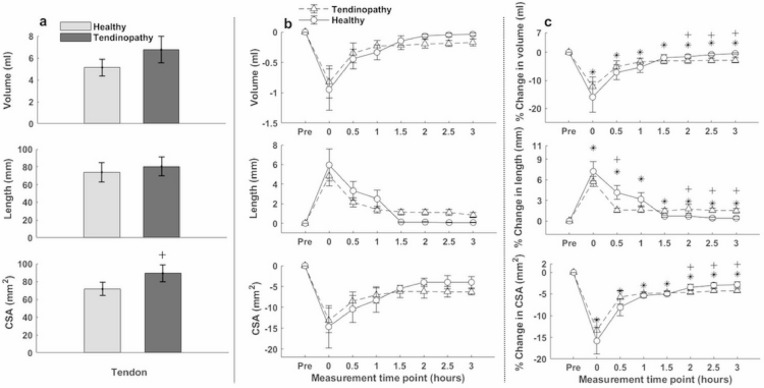
Time course of recovery of free Achilles tendon volume, length, and cross-sectional area (CSA) following eccentric exercise. (**a**) Mean resting (pre-exercise) values of volume, length, and CSA for tendinopathic (dark gray) and healthy (light gray) tendons. (**b**) Mean absolute values, defined as the difference between the values at each measurement time point and the corresponding resting values, of free tendon volume, length, and CSA prior to eccentric exercise (Pre) and during the recovery period (0-3 hours) in tendinopathic (dashed line, triangle) and healthy tendons (solid line, circle). (**c**) Mean percentage change relative to the pre-exercise (resting) value for tendon volume, length, and CSA measured prior to eccentric exercise and throughout the recovery period. *Significantly different from the pre-exercise baseline measurement for both healthy and tendinopathic tendons (p < 0.05). +Significantly different between tendinopathic and healthy tendons (p < 0.05). Within-tendon differences between consecutive post-exercise time points are described in the text and were not individually annotated in the figure to preserve visual clarity. Data are expressed as mean ± one standard error of the mean (n = 20).

The resting, absolute, and percentage change values of free AT volume, length, and CSA are depicted in Fig. 3. At the pre-exercise (resting) time point, there were no significant group differences in free AT volume (tendinopathy: 6.8 ± 3.9 ml, healthy: 5.1 ± 2.5 ml, t(18) = −1.78, *p* = 0.08, d = 0.52) or length (tendinopathy: 80 ± 34 mm, healthy: 74 ± 35 mm, t(18) = −0.42, *p* = 0.67, d = 0.17). However, the mean resting CSA was significantly higher for the tendinopathic compared to healthy tendons (tendinopathy: 89 ± 30 mm², healthy: 72 ± 23 mm², t(18) = −4.86, *p* < 0.001, d = 1.42) Fig. 3A. Eccentric exercise induced an immediate reduction in free tendon volume and CSA, along with an increase in tendon length in both healthy and tendinopathic tendons (*p* = 0.004–0.009). Significant interaction effects between tendon type and measurement time points were observed for percentage changes in volume, length, and CSA (F₇,₁₂ = 3.539, *p* = 0.02, ηp² = 0.41; F₇,₁₂ = 2.93, *p* = 0.04, ηp² = 0.35; F₇,₁₂ = 19.76, *p* < 0.001, ηp² = 0.71 respectively) Fig. 3B&C.

Planned contrasts revealed that for both healthy and tendinopathic tendons, tendon volume and CSA were significantly lower than the pre-exercise baseline at all post-exercise measurement time points (*p* = 0.002–0.041), whereas tendon length was significantly greater than baseline (*p* = 0.003–0.038). In tendinopathic tendon, the percentage changes in volume, length, and CSA plateaued at 1-hour post-exercise, with no significant changes observed at subsequent measurement time points (*p* = 0.21–0.84). In healthy tendon, however, the percentage changes in volume and CSA gradually increased, and the percentage change in length gradually decreased up to 3 h during recovery (*p* = 0.01–0.03). Furthermore, the planned contrasts demonstrated that in tendinopathic tendon, the percentage changes in volume and CSA were significantly lower, and the percentage change in length was significantly greater compared to healthy tendon at the 2, 2.5, and 3-hour measurement time points (*p* = 0.003–0.021) Figure 3B&C. An additional analysis of free AT mid-portion CSA (defined as 40–70% of the free AT length^31^ showed a similar pattern of response and recovery (Supplementary Figure S1).

### Modeling of the free AT recovery

**Fig. 4:**
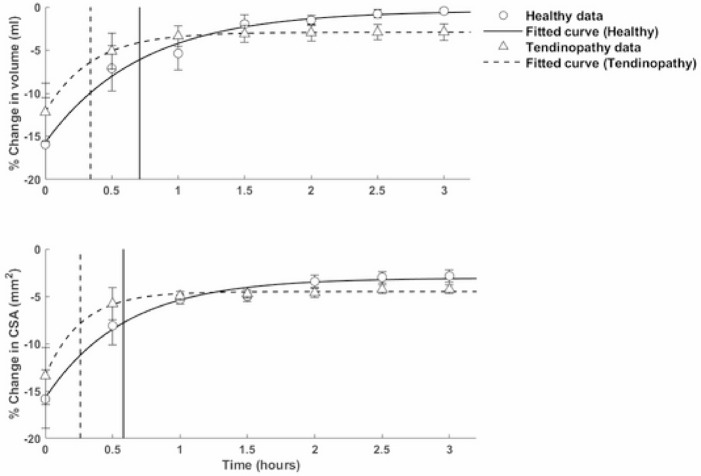
Exponential fit to experimental mean percentage change of free Achilles tendon volume and cross-sectional area (CSA) during the recovery period data for tendinopathic (dashed line, triangle) and healthy (solid line, circle) tendons. The vertical lines intersecting the x-axis represent the time constants (i.e., time required to achieve 63% of initial value) for tendinopathic (dashed line) and healthy tendons (solid line) respectively. Data are expressed as mean ± one standard error of the mean (n = 20).

Exponential curve fits of the recovery time course of tendon volume and CSA Fig. 4 revealed a more rapid early recovery in the tendinopathic group compared with healthy tendons, as reflected by shorter time constant b values [Table 1].

**Table 1:** Tendon recovery modelling parameters.

Parameter	Tendon	Coefficients (a, b, c)	RMSE	*R* ^2^	Time constant (min)
Volume	Tendinopathy	9.28, 0.34, −12.19	0.05	0.99	21
	Healthy	15.32, 0.71, −15.72	0.78	0.98	43
CSA	Tendinopathy	8.90, 0.26, −13.39	0.24	0.99	14
	Healthy	12.67, 0.58, −15.72	0.54	0.99	35

Exponential function, *f(t) = a × (1 - exp(-t/b)) + c*; *a*, *b*, and *c* are coefficients; RMSE, root mean square error; R^2^, coefficient of determination.

## Discussion

This study examined the impact of an acute bout of eccentric exercise, comprising six sets of 15 heel drops, on post-exercise recovery of free AT volume, length, and CSA in individuals with unilateral mid-portion Achilles tendinopathy and matched healthy controls. Both tendinopathic and healthy tendons exhibited significant immediate post-exercise reductions in volume and CSA, accompanied by an increase in tendon length. Tendinopathic tendons demonstrated a faster initial return toward pre-exercise values of volume, length, and CSA, as reflected by shorter recovery time constants, compared with healthy tendons. However, despite this rapid early response, tendinopathic tendons reached an early plateau after approximately one hour and exhibited significantly smaller magnitudes of restoration toward pre-exercise morphology than healthy tendons during the 2–3-hour post-exercise period. Neither tendon type returned to pre-exercise values of volume, length, and CSA within the three-hour recovery window. This pattern of rapid initial recovery followed by an early plateau in tendinopathic tendons highlights disruptions in normal tendon recovery dynamics. These altered recovery dynamics may be related to pathological changes commonly observed in tendinopathic tendons, including collagen disorganization, increased proteoglycan content, and elevated water content within the extracellular matrix^38^. Such alterations may modify tendon permeability and fluid redistribution during loading and recovery, thereby influencing the poroelastic behavior of the tendon.

The mean resting free tendon CSA was significantly larger (on average by 25%) in tendinopathic tendon compared to the healthy tendon which falls within the reported range of 23–60% in previous studies^14,29,30,37^. The tendinopathy group also had a higher free AT volume at rest compared to the healthy tendon which only approached statistical significance (*p* = 0.08). These findings are primarily attributed to the pathological alterations in tendon structure and composition in individuals with tendinopathy^38^.

Following acute eccentric exercise, tendinopathic and healthy tendons exhibited comparable immediate morphological responses in both direction and relative magnitude. Both groups demonstrated reductions in tendon volume (− 12.2% vs. − 15.9%) and CSA (− 13.4% vs. − 15.8%), accompanied by increases in tendon length (5.7% vs. 7.2%), with no significant differences between groups. The direction of these immediate changes in free AT morphology is consistent with previous studies reporting acute reductions in tendon thickness^18,19,24^, water content^25^, and CSA^18^ in both healthy and tendinopathic tendons following a single bout of eccentric exercise. It is, however, noted that Obst et al.^20^, who used a lower loading dose (three sets of 15 heel drops) than the present study, reported no significant changes in tendon volume, length, or CSA at rest immediately following eccentric exercise in healthy tendons, suggesting that there may be a threshold dose required to elicit morphological change. The observed reduction in absolute and relative tendon volume and CSA, together with the concomitant increase in free tendon length immediately following eccentric exercise in tendinopathic and healthy tendons may reflect deformation of the tendon matrix and fluid displacement from the tendon core toward the peri-tendinous space^14,25,30^. This fluid displacement may be due to the release of water molecules from glycosaminoglycans, induced by the electric potential generated through cyclic loading, known as streaming potential^39^, and/or from vascular mechanisms (i.e., cyclic loading-induced reduction in tendon blood volume)^40^. These fluid dynamics likely play a critical role in tendon mechanotransduction and homeostasis, facilitating tendon nutrition and may underpin the clinical efficacy of eccentric exercise as an intervention for patients with mid-portion Achilles tendinopathy.

Tendinopathic tendons exhibited shorter recovery time constants for volume and CSA than healthy tendons (21 and 14 min vs. 43 and 35 min, respectively). Notably, the recovery time constants observed for healthy ATs in the present study are substantially shorter than those reported previously for healthy tendon thickness (~ 2.5 h^19^ and ~ 6.5 h^28^. This discrepancy is likely attributable to differences in the morphological outcome measures and loading regime. Whereas prior studies quantified localized tendon thickness at a single anatomical site (typically ~ 2 cm proximal to the calcaneal insertion), the present study assessed whole free AT volume and CSA, which may reflect more rapid fluid redistribution across the tendon rather than localized structural recovery. Differences in loading protocols may also contribute to the disparity in findings between studies, including contraction mode (eccentric-only vs. combined concentric–eccentric calf raises) and external load (bodyweight only vs. additional external resistance of 20–250% bodyweight)^19,28^. The more rapid recovery observed in tendinopathic tendons following eccentric exercise, reflected by shorter recovery time constants, is consistent with prior studies that report increased fluid mobility and altered intratendinous pressure responses under load in pathological tendons^6,13^. Furthermore, evidence from in vivo studies indicate greater load-induced reduction in tendon CSA, volume, and thickness in tendinopathic tendons compared with healthy tendons, consistent with enhanced fluid exudation and altered poroelastic behavior^14^. The present findings extend this work by suggesting that the same pathological characteristics that permit greater fluid loss during loading (e.g., greater matrix permeability) may also facilitate faster fluid redistribution back into the tendon during early recovery, giving the appearance of accelerated recovery despite underlying structural compromise.

Although tendinopathic tendons exhibited a rapid initial recovery following eccentric exercise, this response plateaued after approximately one hour, with morphological recovery remaining significantly lower than that observed in healthy tendons during the subsequent recovery period. Further, at 3-hours post-exercise, the proportion of full recovery achieved was approximately 97% and 76% for tendon volume, 94% and 74% for tendon length, and 82% and 68% for CSA in healthy and tendinopathic tendons, respectively. Reabsorption of exuded fluid is likely governed by time-dependent interactions between intratendinous pressure gradients and matrix permeability^6,13^. Higher permeability in tendinopathic tendons may facilitate rapid fluid re-entry immediately following unloading; however, this response may not be sustained due to a reduced pressure gradient compared with healthy tendons. While healthy tendons achieved near-full recovery (> 90%) following a therapeutic dose of eccentric exercise within the 3-hour recovery window in the present study, a longer recovery duration would be expected for tendinopathic tendons or following more prolonged or higher loading doses, such as those associated with endurance running or football competition^12,26^. Although these between-group differences reached statistical significance, their magnitude was modest and should therefore be interpreted with appropriate caution.

Delayed recovery of tendon morphology following loading may indicate that tendinopathic tendons require a longer time to restore their internal mechanical and fluid balance. If recovery between loading cycles is incomplete, repeated loading during daily activities or rehabilitation exercises may lead to the accumulation of residual deformation within the tendon, potentially contributing to the persistence or progression of tendon pathology. These altered recovery dynamics may therefore have important implications for load management during rehabilitation, suggesting that individuals with Achilles tendinopathy may require recovery periods between repeated loading bouts that exceed the 3-hour window investigated in this study.

An alternative interpretation of the present findings should also be considered. Because tendinopathic tendons typically exhibit increased CSA and water content at rest^6,14,25^, the reductions in tendon volume and CSA observed immediately following loading may temporarily shift tendon morphology toward values more similar to those of healthy tendons. During the recovery phase, morphology may gradually return toward its baseline state. This interpretation is consistent with the known effects of mechanical loading on tendon fluid redistribution and aligns with evidence that long-term loading-based rehabilitation can promote structural improvements in tendinopathic tendons^41^.

Several limitations should be considered when interpreting the findings of this study. First, the study was restricted to examining whole free tendon volume, length, and CSA. Given that the free tendon exhibits region-specific changes in morphological properties during^14,31^ and after loading^20^, the precise region-specific morphological behavior of the free tendon immediately following eccentric exercise and throughout the recovery period remains unknown. Second, the study’s focus on free tendon morphological recovery was limited to a 3-hour window, with measurements taken at 0.5-hour intervals post-exercise. To gain a more comprehensive understanding of tendon recovery dynamics, future research should incorporate more frequent measurements and extend the recovery period beyond 3 h to quantify the timeframe for full recovery. Additionally, 3D ultrasound is limited in its ability to detect the thickness of the tendon paratenon, epitenon, and peri-tendinous space. As a result, the tendon CSA measurements in this study were confined to the tendon core, preventing an assessment of fluid redistribution from the tendon core to the peri-tendinous space immediately after eccentric exercise, as well as the reverse fluid shift during recovery. This limitation could be addressed in future studies by using higher-resolution imaging techniques, such as MRI. Finally, this study employed a widely adopted eccentric exercise protocol for mid-portion Achilles tendinopathy (6 sets of 15 repetitions of heel drops)^32^, and the participant sample consisted exclusively of male adults with unilateral mid-portion Achilles tendinopathy. Thus, caution is warranted when generalizing these findings to other eccentric loading protocols, female populations, other forms of AT pathology (e.g., insertional Achilles tendinopathy), and different tendons, such as the patellar tendon.

In conclusion, following eccentric exercise, both tendinopathic and healthy tendons demonstrated comparable immediate reductions in volume and CSA, along with an increase in tendon length, likely reflecting deformation of the tendon matrix and fluid displacement from the tendon core toward the peri-tendinous space. During the 3-hour recovery phase, tendinopathic tendons exhibited a rapid initial restoration of volume, length, and CSA; however, this recovery plateaued after one hour and remained significantly lower than that observed in healthy tendons within the 2–3-hour post-exercise window. Despite distinct recovery trajectories, neither tendon fully regained its pre-exercise morphological characteristics within the 3-hour recovery period. Overall, these findings suggest that normal tendon morphological recovery following eccentric exercise is compromised in tendinopathic tendons, likely due to pathological alterations at the cellular, molecular, and structural levels associated with tendinopathy that influence poroelastic behavior.

## Supplementary Information

Below is the link to the electronic supplementary material.

Supplementary Material 1

## Data Availability

The datasets generated and/or analyzed during the current study are available from the corresponding author on reasonable request.
