# Supplementary material for: Tendinopathy alters the time course of free achilles tendon morphological recovery following eccentric exercise
Source: Sci Rep. 2026 Jun 10;16:24323. doi: 10.1038/s41598-026-55189-2 (PMC13448716; doi:10.1038/s41598-026-55189-2)
Supplement: Supplementary file 1 [file 41598_2026_55189_MOESM1_ESM.docx]

**Supplementary Figure S1. Time course of recovery of Achilles tendon mid-portion cross-sectional area (CSA) following eccentric exercise.** (a) Absolute changes in mid-portion CSA relative to the pre-exercise (resting) value measured prior to eccentric exercise (Pre) and throughout the recovery period (0–3 hours) in tendinopathic (dashed line, triangles) and healthy tendons (solid line, circles). (b) Percentage change in mid-portion CSA relative to the pre-exercise (resting) value measured prior to eccentric exercise and during the recovery period. *Significantly different from the pre-exercise baseline measurement for both healthy and tendinopathic tendons (p < 0.05). +Significantly different between tendinopathic and healthy tendons (p < 0.05). Data are presented as mean ± standard error of the mean (n = 20).
